# Differential effects of Cu^2+^ and Fe^3+^ ions on in vitro amyloid formation of biologically-relevant α-synuclein variants

**DOI:** 10.1007/s10534-020-00234-4

**Published:** 2020-03-13

**Authors:** Emma Lorentzon, Ranjeet Kumar, Istvan Horvath, Pernilla Wittung-Stafshede

**Affiliations:** 1 Department of Biology and Biological Engineering, Chalmers University of Technology, Gothenburg, Sweden; 2 Present Address: Department of Chemistry and Molecular Biology, University of Gothenburg, Gothenburg, Sweden

**Keywords:** α-Synuclein, Parkinson’s disease, Metal ions, Acetylation, Thioflavin T, Amyloid formation

## Abstract

Alterations in metal ion homeostasis appear coupled to neurodegenerative disorders but mechanisms are unknown. Amyloid formation of the protein α-synuclein in brain cells is a hallmark of Parkinson’s disease. α-Synuclein can bind several metal ions in vitro and such interactions may affect the assembly process. Here we used biophysical methods to study the effects of micromolar concentrations of Cu^2+^ and Fe^3+^ ions on amyloid formation of selected α-synuclein variants (wild-type and A53T α-synuclein, in normal and N-terminally acetylated forms). As shown previously, Cu^2+^ speeds up aggregation of normal wild-type α-synuclein, but not the acetylated form. However, Cu^2+^ has a minimal effect on (the faster) aggregation of normal A53T α-synuclein, despite that Cu^2+^ binds to this variant. Like Cu^2+^, Fe^3+^ speeds up aggregation of non-acetylated wild-type α-synuclein, but with acetylation, Fe^3+^ instead slows down aggregation. In contrast, for A53T α-synuclein, regardless of acetylation, Fe^3+^ slows down aggregation with the effect being most dramatic for acetylated A53T α-synuclein. The results presented here suggest a correlation between metal-ion modulation effect and intrinsic aggregation speed of the various α-synuclein variants.

**Electronic supplementary material:**

The online version of this article (10.1007/s10534-020-00234-4) contains supplementary material, which is available to authorized users.

## Introduction

A unifying molecular event in neurodegenerative disorders is the aberrant self-assembly of proteins into amyloid fibers with a hallmark cross-β-sheet arrangement. Parkinson’s disease (PD) is the second most common neurodegenerative disorder (after Alzheimer’s disease) and the most common movement disorder. PD is characterized by widespread deterioration of subcortical structures of the brain, especially dopaminergic neurons in the substantia nigra (Chen et al. 2014). Conformational changes resulting in aggregation of the intrinsically-disordered protein α-synuclein (αSyn) into amyloid fibers is directly related to PD (Winner et al. 2011; Galvin et al. 1999). Many amyloidogenic proteins (including αSyn) can bind metals in vitro and systemic as well as tissue imbalances of metal levels are often found to be strongly associated with neurodegenerative disorders including PD (Fink 2006). This implies that metal ions may play pivotal roles in progression of PD and other neurodegenerative disorders (Bush 2000; Bjorklund et al. 2019; Aaseth et al. 2018). Notably, the brain has a high demand for metal ions and, as such, it can viewed as an organ that concentrates metal ions (Bush 2000; Valiente-Gabioud et al. 2012).

For PD, it has been found that copper levels are decreased in brain tissue but increased in fluids, whereas for iron, the levels are increased in brain cells (Bush 2000). The differences noted vary between brain regions (Bjorklund et al. 2019; Aaseth et al. 2018). Also other metal ion levels are altered in PD and, for example, occupational exposure to high manganese levels have been linked to Parkinsonism, a disorder similar to PD (Bjorklund et al. 2019; Aaseth et al. 2018). Of metal ions interacting with αSyn, copper ions display the strongest affinity (nanomolar range) (Binolfi et al. 2010). Copper (Cu) in both redox states (oxidized = Cu^2+^; reduced = Cu^+^) can bind to αSyn (Camponeschi et al. 2013; De Ricco et al. 2015a) and structural features, binding sites, and affinities for the interaction between αSyn and Cu^2+^, as well as Cu^+^, have been the focus of many in vitro spectroscopic investigations (Camponeschi et al. 2013; De Ricco et al. 2015a; Binolfi et al. 2006, 2011; Carboni and Lingor 2015). Whereas the high affinity Cu^2+^ site involves backbone nitrogen atoms in residues Met1 and Asp2, the dominant Cu^+^ binding site involves the side chain sulfurs of Met1 and Met5 (Moriarty et al. 2014a; Miotto et al. 2015). Also iron in both redox states (oxidized = Fe^3+^; reduced = Fe^2+^) can bind to αSyn but with micromolar affinity [differs between redox states, and between published reports (Peng et al. 2010; Davies et al. 2011a)] and the αSyn interaction site involves residues 119 to 124 (_119_DPDNEA_124_) in the C-terminal part (Abeyawardhane and Lucas 2019). Many metal ions can accelerate αSyn amyloid formation, but Cu^2+^ has the largest such effect in vitro (Montes et al. 2014; Davies et al. 2016). However, parallel comparisons of metal-ion effects on αSyn aggregation, at biologically-relevant metal ion concentrations and for biologically-relevant αSyn variants (such as with N-terminal acetylation), are lacking.

Although biological systems are complex and many factors must be taken into consideration (e.g., redox reactions, protein partners, local concentrations, membranes) to understand PD development in patients (Bush 2000; Bjorklund et al. 2019; Aaseth et al. 2018), it is of fundamental importance to reveal intrinsic features of how metal ions affect αSyn amyloid formation. Here we compared the effects of Cu^2+^ and Fe^3+^ on αSyn amyloid formation, using micromolar concentrations of metal ions and a set of PD-relevant αSyn variants, in vitro. The obtained results demonstrate a dependency among metal ion identity, concentration and αSyn variant that appears related to differential αSyn monomer conformation and thereby intrinsic aggregation speed.

## Materials and methods

### Expression and purification of αSyn variants

Human WT and A53T αSyn proteins in non-acetylated forms were expressed heterologously in *Escherichia coli* as reported in Werner et al. (2018). In short, plasmids for WT and A53T αSyn were transformed into BL21 (DE3) (Novagen) cells. The bacteria were first grown to an OD_600_ of 0.6 in Luria broth (LB) containing 100 μg/ml carbenicillin at 37 °C and then induced with 1 mM IPTG (isopropyl β-d-1–thiogalactopyranoside) and grown overnight at 25 °C post induction. The cells were harvested and lysed by sonication on an ice bath in 20 mM Tris–HCl buffer pH 8.0 in the presence of protease inhibitor cocktail (Roche). The lysate after sonication was treated with a universal nuclease (Pierce) for 15 min at room temperature. The lysate was then heat treated at 90 °C in a water bath for 10 min followed by centrifugation at 15,000 × *g* for 30 min. The supernatant was then filtered through 0.2 μm filter and loaded on to a pre-equilibrated 5 ml HiTrap Q FF anion exchange column (GE Healthcare). The αSyn proteins were eluted by a linear gradient with 1 M NaCl in 20 mM Tris–HCl buffer pH 8.0. The eluted protein were run on a 4–12% SDS-PAGE and fractions containing the protein of interest were pooled and concentrated with Amicon Ultra-15 10 K centrifugal filter units (Millipore). The concentrated protein was loaded and retrieved from a pre-equilibrated Hiload 16/600 Superdex 75 pg column (GE Healthcare) with 20 mM Tris–sulfate buffer pH 7.4. For all purified αSyn variants, the sample purity was confirmed by a single band on SDS-PAGE gel, a single elution peak in size exclusion chromatography, and by mass spectrometry. Fractions containing pure protein were pooled and snap frozen in liquid nitrogen and stored at − 80 °C. The concentration of WT and A53T αSyn was determined using ε_280_ = 5960 M^−1^ cm^−1^. Acetylated WT and A53T αSyn proteins were overexpressed by co-transforming the pT7-7 αSyn plasmid with pNatB (a kind gift of D. P. Mulvihill) (Johnson et al. 2013), expressing the yeast *N*-acetyl-transferase NatB gene. After expression, the protein purification procedure was exactly the same as for the non-acetylated αSyn variants, see above. The co-expression process resulted in the isolation of fully amino-terminal acetylated αSyn protein, which was confirmed by mass spectrometry.

As described earlier (Horvath et al. 2018), preparation of (non-acetylated) truncated αSyn (residues 1–97) used a pET3a construct (including a tag with a repressor protein and a His-stretch followed by a Caspase 7 cleavage site) with the gene for truncated αSyn. The plasmid was transformed into BL21(DE3) (Novagen) cells, which were then grown in similar conditions as for WT αSyn. The cells were harvested by centrifugation and re-suspended in Buffer A (20 mM Tris–HCl pH 8, 20 mM imidazole and 8 M urea), then sonicated and centrifuged for 30 min at 15,000×*g*. The supernatant was filtered through a 0.2 µm filter and applied to a 5 ml Hi Trap Ni–NTA column (GE healthcare) pre-equilibrated with Buffer A. After washing the column with 5 column volumes of buffer B (20 mM Tris–sulfate pH 7.4, 20 mM imidazole, 50 mM NaCl), truncated αSyn was eluted with buffer B, with added 250 mM imidazole. The tag was cleaved upon addition of Caspase 7 in the ratio of 1:100 (caspase 7: truncated αS w/w) in the presence of 5 mM TCEP (tris (2-carboxyethyl) phosphine) and incubated overnight at 4 °C. The protein was checked for cleavage and dialyzed overnight to remove excess imidazole. The cleaved protein was loaded onto a 5 mL HiTrap Q FF anion exchange column (GE Healthcare) and eluted by a linear gradient of 1 M NaCl in 20 mM Tris–HCl buffer, pH 8.0. The eluted fractions were pooled, concentrated and loaded onto a Hiload 16/600 Superdex 75 column (GE Healthcare). The protein was eluted in 20 mM Tris–HCl buffer pH 7.4. Fractions containing αSyn were pooled, snap frozen in liquid nitrogen and stored in -80 °C.

### ThT aggregation assay

αSyn amyloid formation reactions were conducted in 96-well half-area transparent bottom plates with a non-binding surface (Corning, CLS3881) with one 2-mm glass bead in each well using a plate reader-incubator instrument (BMG Labtech, Fluostar Optima). The plates were sealed with transparent tape to prevent evaporation and contamination. Measurements performed in TBS (0.05 M Tris–HCl buffer, pH 7.4 with 0.15 M NaCl, 93318 Sigma-Aldrich) buffer in the presence of 20 µM Thioflavin T (ThT, T3516 Sigma-Aldrich) at 37 °C using 200 rpm agitation for 5 min during the 20 min measurement cycles, fluorescence was measured from the bottom side of the plate. All αSyn samples were gel filtered prior to aggregation experiments in order to ensure the starting point is only monomers. In all aggregation measurements, the αSyn concentration was 50 µM. Samples were typically incubated for 60 h and fluorescence measured every 20 min. ThT was excited at 440 nm and emission was recorded at 480 nm. All ThT experiments were performed in quadruplicates at each time, and repeated at least three independent times. For seeded ThT aggregation experiments (in which elongation dominate the kinetics) of WT αSyn as a function of Fe^3+^, preformed αSyn amyloids were added (5 μM monomer concentration) to fresh αSyn monomers (50 μM) and aggregation experiments, as above, were started.

### Atomic force microscopy (AFM)

Aggregated samples from ThT experiments were diluted into MilliQ water (10–20 times) and deposited on freshly cleaved mica. After 10 min, the mica was rinsed with filtered Milli-Q water and dried under a gentle nitrogen stream. Images were recorded on an NTEGRA Prima setup (NT-MDT) using a gold-coated single crystal silicon cantilever (NT-MDT, NSG01, spring constant of ~ 5.1 N/m) and a resonance frequency of ~ 180 kHz. 256 pixel images were acquired with 0.5 Hz scan rate. Images were analyzed using the WSxM 5.0 software (Horcas et al. 2007). For each sample images were collected in at least three different 50 × 50 µm areas, the images shown are representative for each sample.

### Transmission electron microscopy (TEM)

The samples were taken from the incubation mixture from the ThT-aggregation assays after 60–100 h (the samples were considered to be at the end of fibril formation stage), in the presence and absence of metal ions. The samples were placed on glow-charged, formvar coated, carbon-stabilized copper EM grids. Absorption of sample lasted 2 min, followed by staining with 1 percent aqueous phosphotungstate (1% PTA). The grids were then blotted with filter paper and dried before use (Choi et al. 2018). High contrast images were obtained with a TALOS L120C TEM at an accelerating voltage of 120 kV, using a 4 × 4 k CMOS Ceta camera. The magnification was set to 17,500×–300000×.

### Circular dichroism (CD)

CD was measured in the near-UV region (250–700 nm) on Jasco J-810 and Chirascan-CS spectrophotometers using a 1-cm quartz cuvette at 20 ºC. Protein samples (ca 100–150 μM αSyn in the same TBS buffer as in aggregation experiments) were titrated with Cu^2+^, going from 1:0.25 to 1:2 protein:Cu ratios, with approximately 10 min incubation between each addition. Each experiment was repeated three times. The CD data was normalized to mean residue ellipticity.

## Results

We monitored, using the thioflavin T (ThT) fluorescence assay, the kinetics of aggregation of selected αSyn variants (50 μM) as a function of Cu^2+^ and Fe^3+^ ions (in the range 0–400 μM) at pH 7, 37 °C under agitation with a glass bead. Agitation in combination with a glass bead is used to increase the reproducibility of the kinetic data (Giehm et al. 2011). The presence of only αSyn monomers at the starting point was ensured by performing gel filtration chromatography of the samples directly prior to each aggregation experiment. As it is now established that αSyn is acetylated at the N-terminal amino group in vivo (Bartels et al. 2011), we here focused on wild-type (WT) and Ala53Thr (A53T; early onset PD-causing mutation) (Polymeropoulos et al. 1997; Kruger et al. 1998) αSyn variants in both non-acetylated and acetylated states. The acetylated αSyn variants were prepared using an established procedure (Johnson et al. 2013) and modification of the resulting protein was confirmed by mass spectrometry. Representative kinetic aggregation curves for the four proteins alone and upon additions of the metal ions are shown in Figures S1–S3.

### Effect of Cu ions on amyloid formation of αSyn variants

**Fig. 1:**
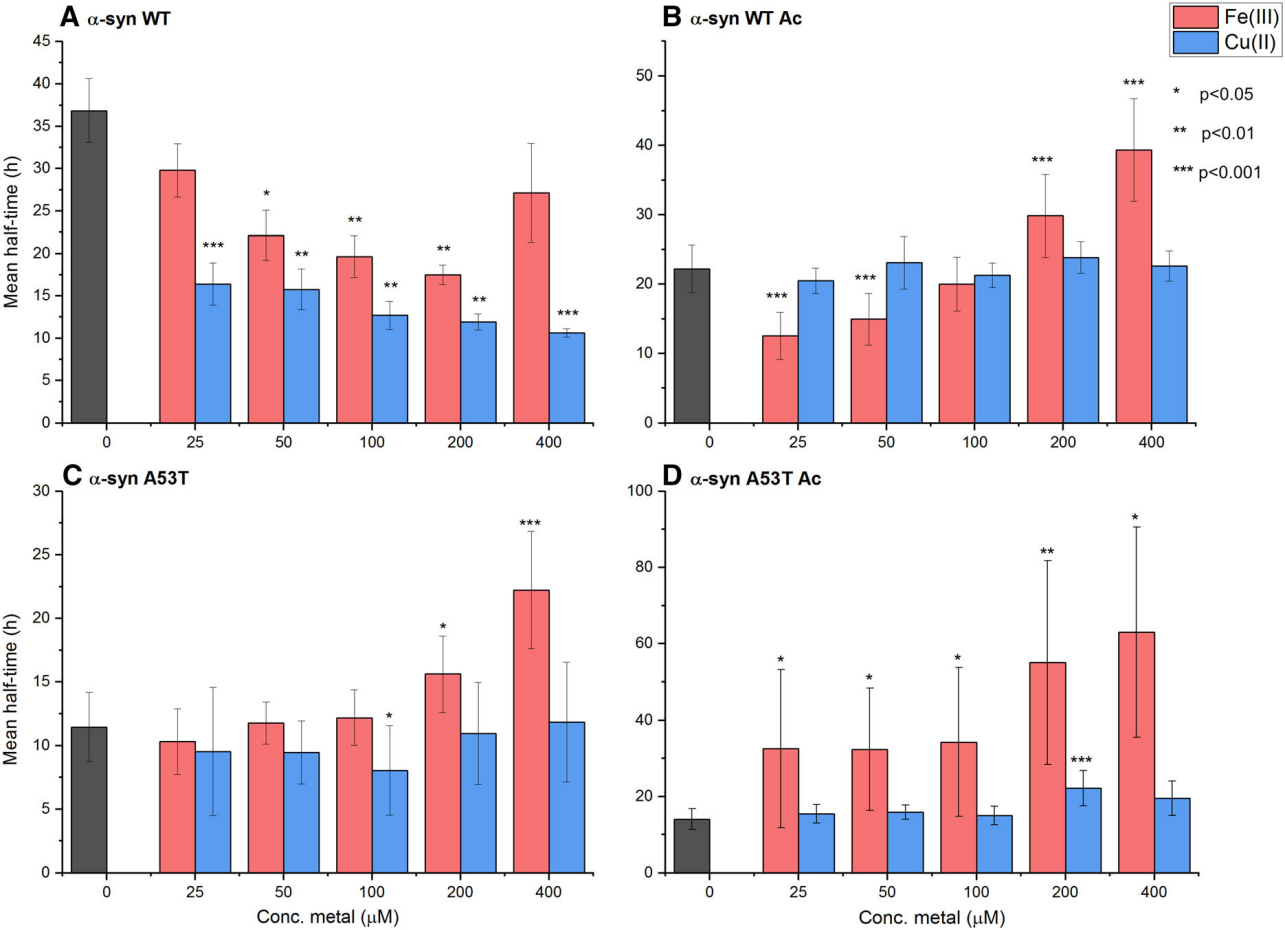
Midpoints of aggregation curves (derived from ThT-detected kinetic data; Figures S2–S3) for various additions of Fe^3+^ (red) and Cu^2+^ (blue) to αSyn variants: **a** WT αSyn. **b** Acetylated WT αSyn. **c** A53T αSyn. **d** Acetylated A53T αSyn. Significance scale according to: *p < 0.05, **p < 0.01, ***p < 0.001. The p values were obtained by the Student’s *t* test method. The shown data is based on three independent experiments with four replica in each. Midpoints are defined as the time when the ThT signal has reached 50% of its final value

As expected (Moriarty et al. 2014a; Montes et al. 2014; Davies et al. 2016), we found Cu^2+^ to promote aggregation of non-acetylated WT αSyn in a concentration dependent manner but these metal ions had no effect on aggregation kinetics of acetylated WT αSyn (Figs. 1, S2). Surprisingly, for non-acetylated A53T αSyn, Cu^2+^ had no effects on the kinetics of aggregation, although the mutation is not involving Cu-binding residues. In similarity with acetylated WT αSyn, and reflecting lack of the high affinity binding site, aggregation of acetylated A53T αSyn was not affected by Cu^2+^ additions.

**Fig. 2:**
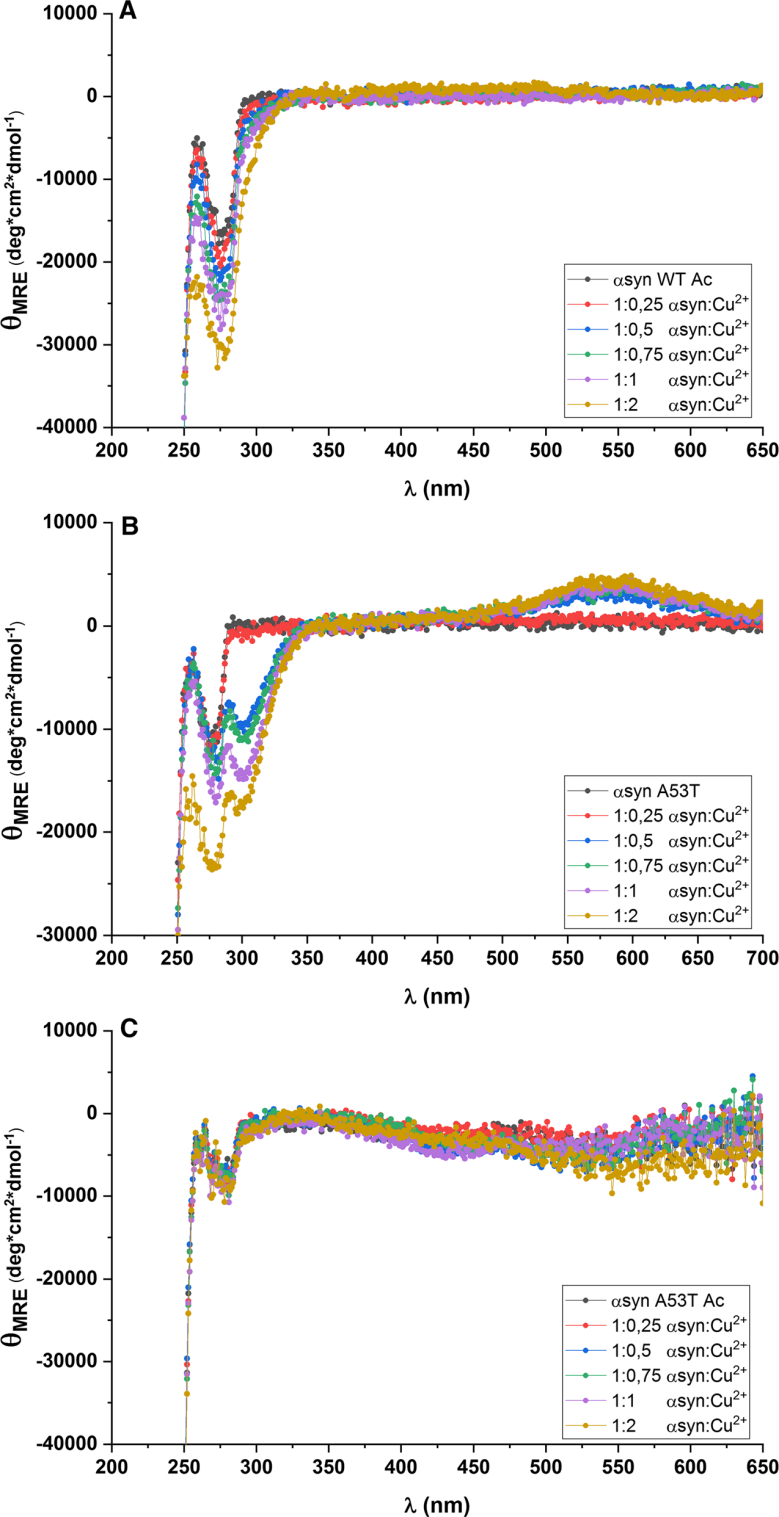
Near-UV CD spectra for αSyn variants upon addition of Cu^2+^ as indicated. **a** Acetylated WT αSyn. **b** A53T αSyn. **c** Acetylated A53T αSyn

To assure that Cu^2+^ binds to A53T αSyn at our conditions, despite the lack of effect on aggregation kinetics, we turned to near-UV circular dichroism spectroscopy (CD). Cu^2+^ binding to the N-terminal Cu^2+^ site in αSyn can be detected via a negative CD signal around 300 nm (charge transfer transition from metal center to an imidazole group or deprotonated peptide nitrogen) and a positive CD signal at 600 nm (d–d transition) (Binolfi et al. 2006, 2010; Rasia et al. 2005). In Fig. 2, we show that non-acetylated A53T αSyn binds Cu^2+^, in an apparent similar coordination to WT αSyn (Binolfi et al. 2010), but none of the two acetylated variants bind Cu^2+^. Thus, Cu^2+^ binds efficiently to A53T αSyn but this interaction does not affect aggregation kinetics. To test if the lack of Cu^2+^ effect on aggregation relates to intrinsic speed of aggregation, as A53T αSyn aggregates faster than WT αSyn, we investigated truncated αSyn (contains only residues 1–97) that aggregates even faster than A53T αSyn. The CD and ThT data in Fig. S4 demonstrate that truncated αSyn interacts with Cu^2+^ like WT and A53T αSyn, but (in similarity to A53T αSyn) this interaction has no effect on aggregation kinetics of truncated αSyn.

### Effect of Fe ions on amyloid formation of αSyn variants

Like Cu^2+^, Fe^3+^ speeds up aggregation of non-acetylated WT αSyn, but with acetylation, Fe^3+^ additions gives a complex trend (Figs. 1, S3). At low Fe^3+^ concentration (25, 50 μM), aggregation of αSyn becomes faster whereas at higher Fe^3+^ concentration (200 and 400 μM) metal binding slows down aggregation of αSyn. In contrast, for A53T αSyn, regardless of acetylation or not, for all concentrations, Fe^3+^ slows down aggregation with the effect being most dramatic for the acetylated form of A53T αSyn (Figs. 1, S3). We note that Fe^3+^ has a tendency to precipitate in pH-neutral solutions (Peng et al. 2010) and we checked on this carefully. At our conditions and incubation times, like in other studies (Abeyawardhane et al. 2018), there was no precipitation of Fe^3+^ detected. In addition, as a control, we tested the effect of Fe^3+^ ions on the aggregation reaction of truncated αSyn. As the binding site for Fe^3+^ is suggested to be in the C-terminus (Joppe et al. 2019), Fe^3+^ should not affect aggregation of truncated αSyn—unless artifacts were at play. In Figure S5, we show that Fe^3+^ has no effect on truncated αSyn aggregation kinetics. This result supports that modulation of aggregation kinetics of WT and A53T αSyn upon Fe^3+^ additions are consequences of metal binding to the protein.

By the use of seeded αSyn aggregation experiments, where the fiber elongation process dominates, one can assess if an inhibitory effect is due to blockage of amyloid fiber elongation or not (Meisl et al. 2016). Seeding of the aggregation reaction of acetylated WT αSyn (using 10% amyloid seeds) speeds up aggregation, as expected. When Fe^3+^ was added to such seeded reactions, the metal ions had no effect on αSyn aggregation kinetics (Figure S6). Thus, Fe^3+^-mediated inhibition of amyloid formation of acetylated αSyn is not due to effects on elongation but appears linked to nucleation steps (primary or secondary nucleation).

For all the αSyn aggregation reactions in the presence of metal ions, we checked the resulting amyloid fibers by AFM or TEM at the end of the aggregation experiments (Figure S7). In all cases, including the ones where fiber formation was delayed, amyloids are formed and there are no striking differences in amyloid fiber appearances (for this resolution) without and with added metal ions.

## Discussion

To eventually counteract neurodegenerative diseases, we must gain better knowledge of metal-ion interactions that may modulate amyloid formation. Interestingly, αSyn, which aggregates into amyloid fibers in PD, is annotated in uniprot.org as copper-binding and it can also bind other metal ions in vitro (Montes et al. 2014; Davies et al. 2016). Progressive increase of metal ion content in brain tissue is thought to be a consequence of normal aging and may be one factor promoting age-related neurodegeneration (Valiente-Gabioud et al. 2012). Also prolonged exposure to *e.g.* iron, manganese and copper via external sources (such as industrial work exposure) increases the risk for PD and other neurodegenerative diseases (Bjorklund et al. 2019; Aaseth et al. 2018; Valiente-Gabioud et al. 2012). In brain biopsies from PD patients, iron levels are elevated while cellular copper levels appear reduced (Abeyawardhane and Lucas 2019). Nonetheless, extracellular copper levels at the synapse may reach high micromolar concentrations (Kardos et al. 1989) and for PD patients, copper is elevated in serum (Bush 2000). Because iron and copper dys-homeostasis appears directly related to PD, and both metal ions bind to αSyn in vitro, we here compared the effects of micromolar concentrations of Cu^2+^ and Fe^3+^ on the in vitro aggregation of selected, biologically-relevant αSyn variants. Our results may act as a biophysical starting point for more advanced investigations taking biological aspects into account. Although there has been previous reports of the effects of these metal ions on αSyn aggregation, the two ions have not been compared in parallel nor have the consequences of αSyn mutation and acetylation been addressed in combination with these metal ions.

**Fig. 3:**
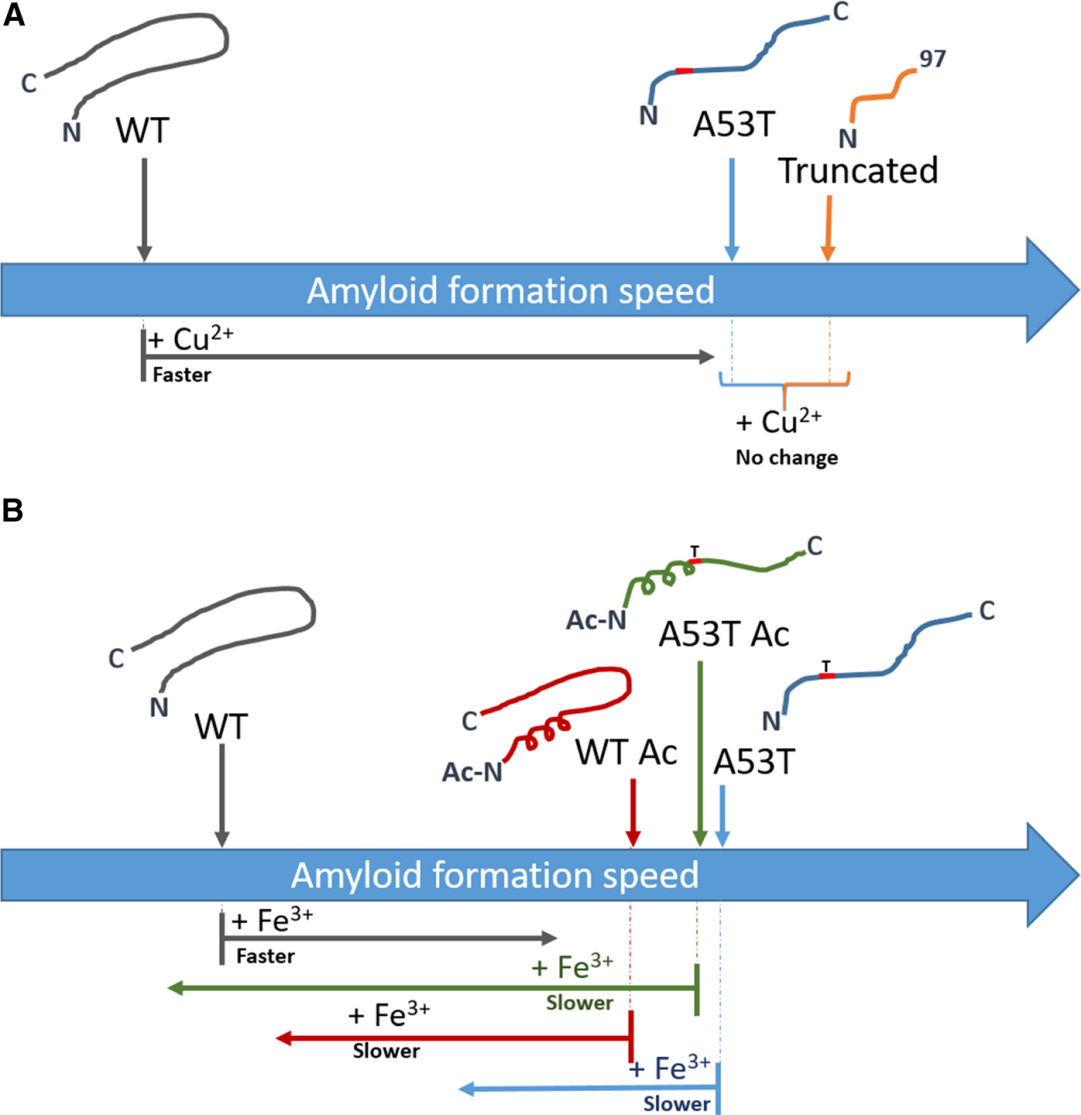
Scheme illustrating the link between metal effects and intrinsic αSyn variant aggregation speed (monomer conformation). **a** Aggregation-promoting effect of Cu^2+^ is only found when intrinsic aggregation is slow, as for WT αSyn. For A53T and truncated (1–97) αSyn, which both aggregates faster, Cu^2+^ has no accelerating effect. **b** Aggregation-modulating effect of Fe^3+^ depends on intrinsic aggregation speed. Whereas WT αSyn is accelerated by Fe^3+^, the faster aggregating variants acetylated WT αSyn, A53T αSyn and acetylated A53T are instead slowed down by Fe^3+^. The intrinsic αSyn aggregation speed of variants can be linked to their individual monomer conformational landscape, with WT αSyn being more compacted (involving inhibitory interactions between N- and C-termini) than A53T αSyn and acetylation results in transient helicity in the N-terminal part (see text)

In accord with several reports, we found Cu^2+^ to accelerate non-acetylated WT αSyn aggregation (Montes et al. 2014; Davies et al. 2016; Uversky et al. 2001) whereas with acetylation, the reaction was not affected by Cu^2+^ (Moriarty et al. 2014b; Mason et al. 2016). Surprisingly, for A53T αSyn, regardless of acetylation or not, Cu^2+^ additions did not affect aggregation kinetics although our CD experiments, as expected, showed that Cu^2+^ binds to non-acetylated A53T αSyn like it does to WT αSyn. For the acetylated forms, WT and A53T αSyn, no CD signals appeared upon Cu^2+^ additions, indicative of lack of metal-ion binding. Acetylation blocks the N-terminal high-affinity Cu^2+^ site, but His50 (and C-terminal residues with weak affinity to Cu^2+^) remains a putative Cu^2+^-binding site. However, if Cu^2+^ would bind around His50, such an interaction should give rise to a CD signal (Tian et al. 2019) and thus we conclude that for our acetylated αSyn variants at our conditions, the affinity of Cu^2+^ must be less than 100 μM. That Cu^2+^ binding does not affect aggregation kinetics of non-acetylated A53T αSyn may be related to the intrinsically faster aggregation speed of this variant (Figure S1). The faster aggregation speed of A53T αSyn (as compared to WT) has been linked to fewer long-range contacts in the mutant’s monomeric state (Bertoncini et al. 2005). Thus, Cu^2+^ binding to WT αSyn may change the monomer conformation towards that of A53T αSyn, with the consequence of faster aggregation (Fig. 3). For A53T αSyn, already in a more extended conformation, Cu^2+^ binding may not affect the conformational landscape of the monomer further, and thus Cu^2+^ binding does not affect kinetics of aggregation. In accord with this reasoning, for truncated αSyn, that aggregates even faster than A53T αSyn, Cu^2+^ binding had no effect on the aggregation kinetics (Figure S4).

It has been reported that millimolar Fe^3+^ speeds up non-acetylated WT αSyn aggregation (Uversky et al. 2001) but the effect of Fe^3+^ on aggregation kinetics of acetylated αSyn has not been tested. One study tested the effects of Fe^3+^ and Fe^2+^ on resulting amyloid fiber structures of acetylated WT αSyn, but no kinetics were reported (Abeyawardhane et al. 2018). Our data show that when probed in the micromolar range, Fe^3+^ accelerates aggregation of non-acetylated WT αSyn, but for acetylated WT and A53T αSyn (with and without acetylation) the effect of Fe^3+^ is to slow down the aggregation kinetics. We rationalize this by differences in conformations of the monomeric variants that, in turn, relate to intrinsic aggregation speeds. As mentioned, A53T aggregates faster than WT αSyn (Figure S1). When WT αSyn is acetylated, it aggregates faster, but acetylation does not matter much for intrinsic aggregation speed of A53T αSyn (Figure S1). While the A53T αSyn variant has less long-range contacts (Bertoncini et al. 2005), N-terminal αSyn acetylation results in increased local helical content (Kang et al. 2012). Both these alterations thus allows for faster aggregation kinetics, with the mutation having the largest effect. Therefore, we speculate that Fe^3+^ binding to non-acetylated WT αSyn alters the monomer conformation towards that of the mutant (i.e., more extended structure) and this speeds up aggregation. In contrast, for acetylated WT and both forms of A53T αSyn, Fe^3+^ binding instead changes the conformations (towards more compact forms) such that aggregation becomes slower (illustrated in Fig. 3).

In vivo, many factors, such as metal-induced redox reactions (Abeyawardhane and Lucas 2019; Miotto et al. 2014; Davies et al. 2011b) and metal-binding proteins (Montes et al. 2014; McLeary et al. 2019), have to be taken into account (Bush 2000). For example, it was recently shown that, when bound to αSyn, copper could cycle between oxidized and reduced forms (Miotto et al. 2014; Davies et al. 2011b; Wang et al. 2010) and such copper-bound αSyn was able to act as a ferri-reductase using the copper ion as a catalytic redox center (Brown 2013; De Ricco et al. 2015b). In another study, it was shown that the copper transport protein Atox1, which transports Cu^+^ in the cytoplasm, could form a complex with αSyn (regardless of acetylation) and this interaction blocked αSyn aggregation (Horvath et al. 2019). It is also possible that the resulting αSyn amyloid fibers, and intermediate species (oligomers), differ in the presence or absence of metal ions. One study showed that in the presence of Cu^2+^, the formed αSyn amyloid fibers appeared more neurotoxic than in the absence of Cu^2+^ (Choi et al. 2018). To resolve the connection between metal ion metabolism and neurodegeneration, many additional studies, spanning from biophysics in vitro (as here) to cellular models and living systems, are desired.

## Electronic supplementary material

Below is the link to the electronic supplementary material.

Supplementary file1 (PDF 1468 kb)
