# Supplementary material for: Differential effects of Cu2+ and Fe3+ ions on in vitro amyloid formation of biologically-relevant α-synuclein variants
Source: Biometals. 2020 Mar 13;33(2):97–106. doi: 10.1007/s10534-020-00234-4 (PMC7295844; doi:10.1007/s10534-020-00234-4)
Supplement: Supplementary file 1 — Supplementary file1 (PDF 1468 kb) [file 10534_2020_234_MOESM1_ESM.pdf]

Supporting Information

Differential effects of Cu^{2+} and Fe^{3+} ions on *in vitro* amyloid formation of biologically-relevant α-synuclein variants

Emma Lorentzon, Ranjeet Kumar, Istvan Horvath, Pernilla Wittung-Stafshede

Content:

Figures S1-S7

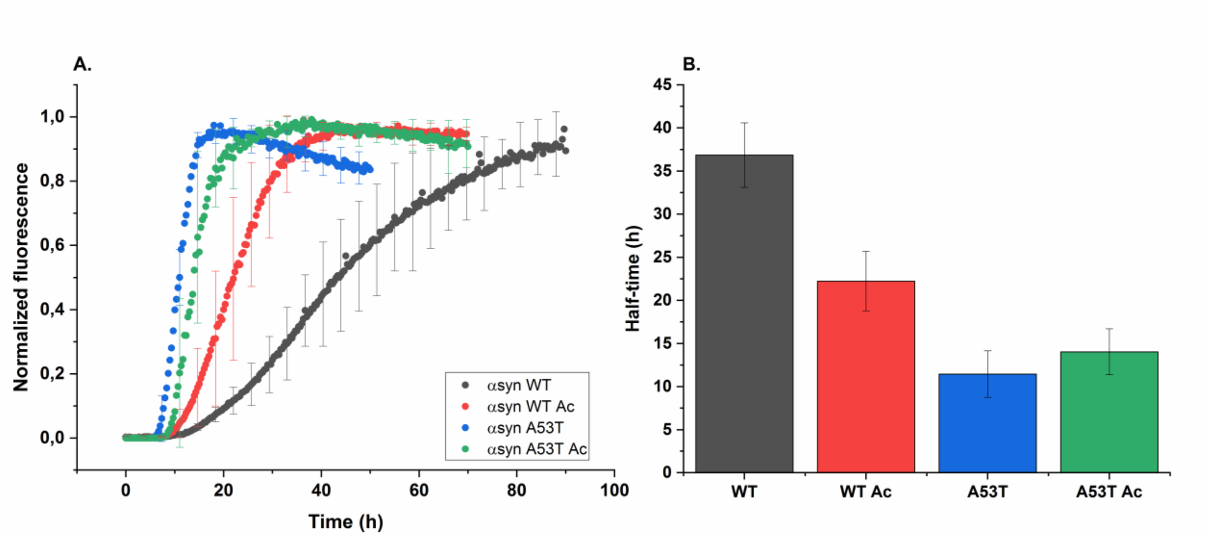

Figure S1. Aggregation kinetics (probed via ThT fluorescence) of the four studied αSyn variants (50 µM). A. Aggregation curves of WT αSyn, A53T αSyn, acetylated WT αSyn and acetylated A53T αSyn. B. Midpoint times for the αSyn variants. Average values and standard deviations were calculated based on at least three repeats (with for replicas in each). Midpoints were defined as the time point where the fluorescence change in each aggregation curve reached 50%.

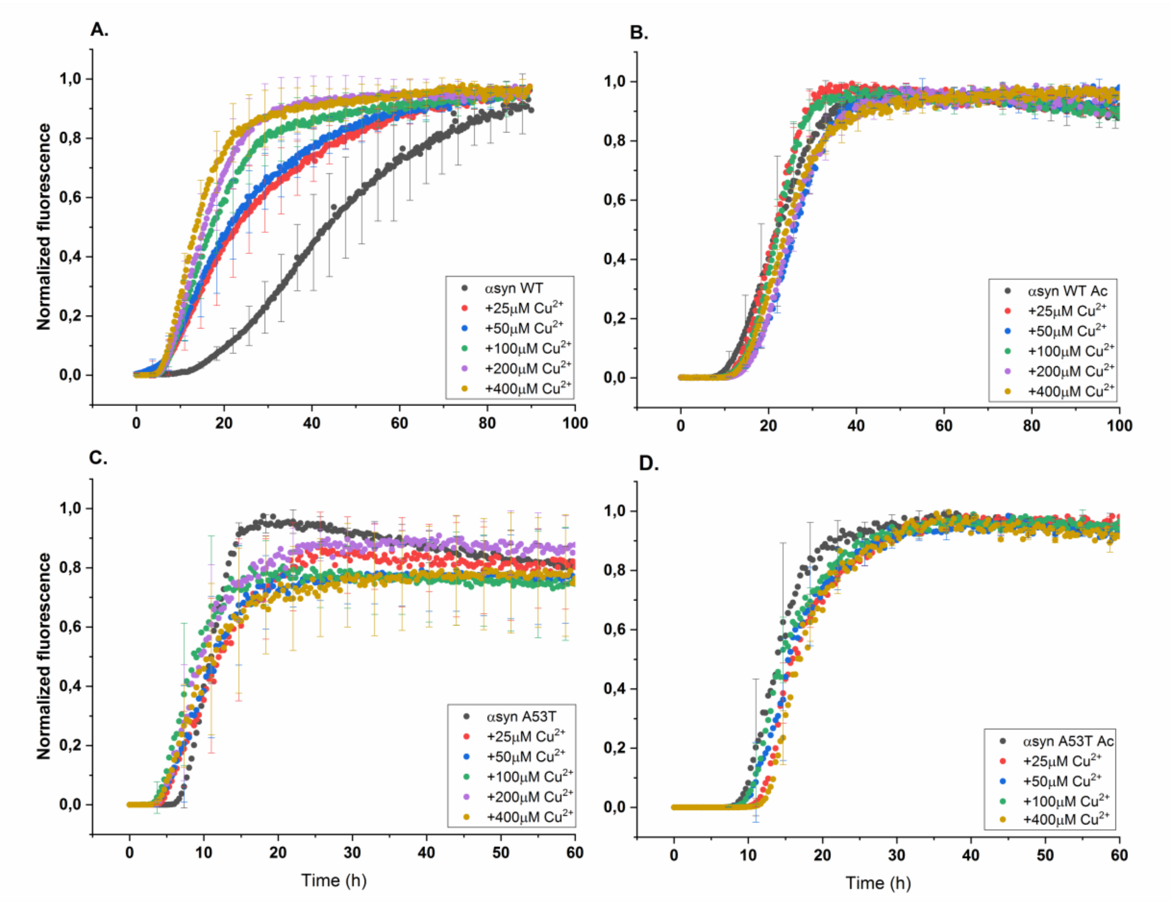

Figure S2. ThT aggregation curves for αSyn variants: non-acetylated WT (A), acetylated WT (B), non-acetylated A53T (C) and acetylated A53T (D) in the absence and presence of various concentrations of Cu^{2+}. Standard deviation was calculated based on at least three repeats (with for replicas in each).

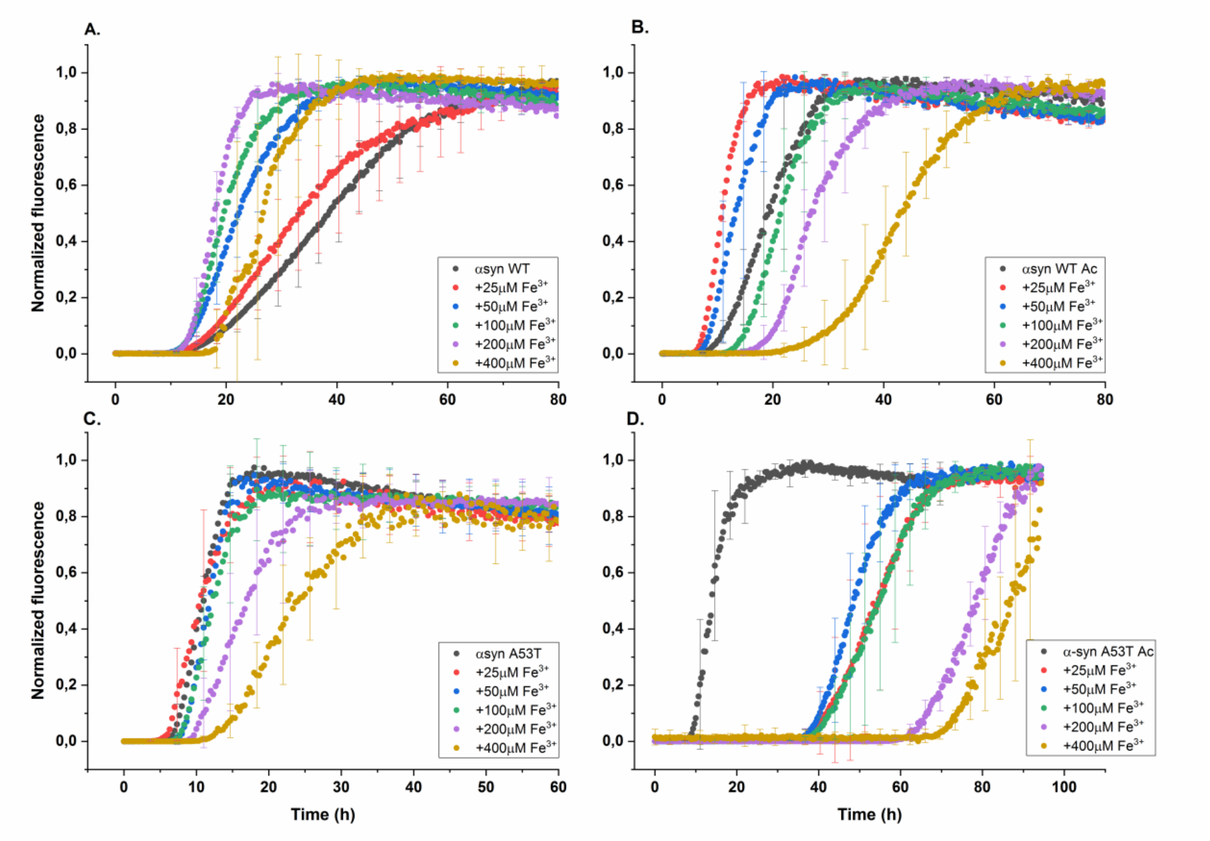

Figure S3. ThT aggregation curves for αSyn variants: non-acetylated WT (A), acetylated WT (B), non-acetylated A53T (C) and acetylated A53T (D) in the absence and presence of various concentrations of Fe^{3+}. Standard deviation was calculated based on at least three repeats (with for replicas in each).

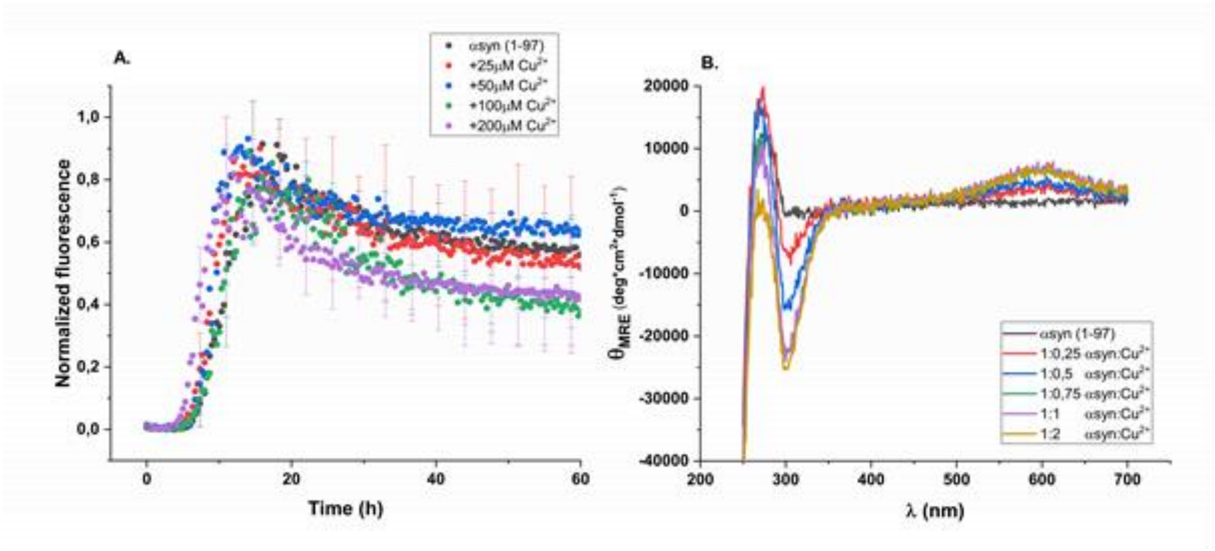

Figure S4. A. Aggregation kinetics for truncated αSyn as a function of Cu^{2+}. B. CD spectra of truncated αSyn upon additions of Cu^{2+}.

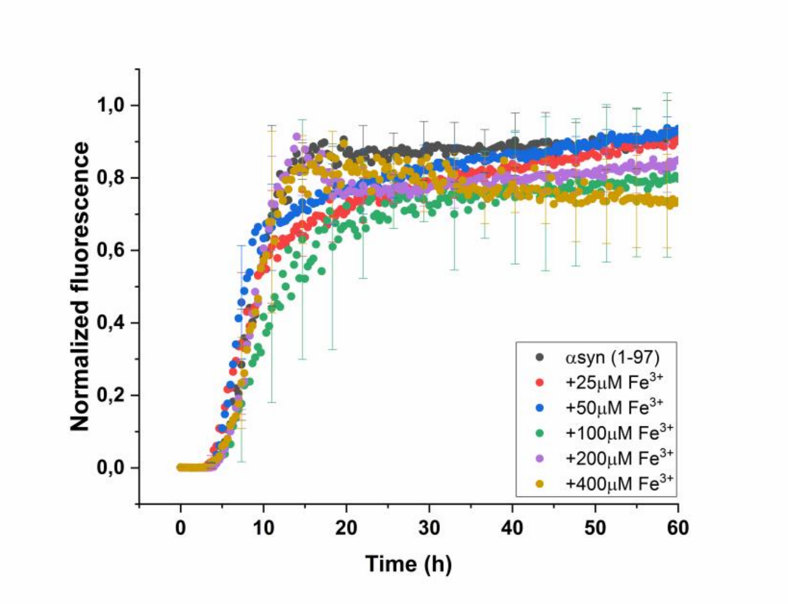

Figure S5. Aggregation kinetics for truncated αSyn as a function of Fe^{3+}.

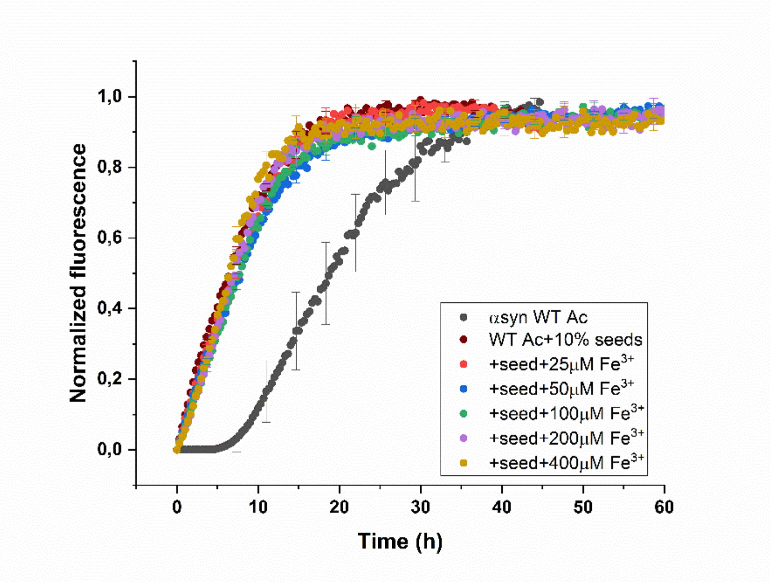

Figure S6. Aggregation reactions of acetylated WT αSyn upon the addition of 10 % pre-formed amyloid seeds, as a function of Fe^{3+} additions.

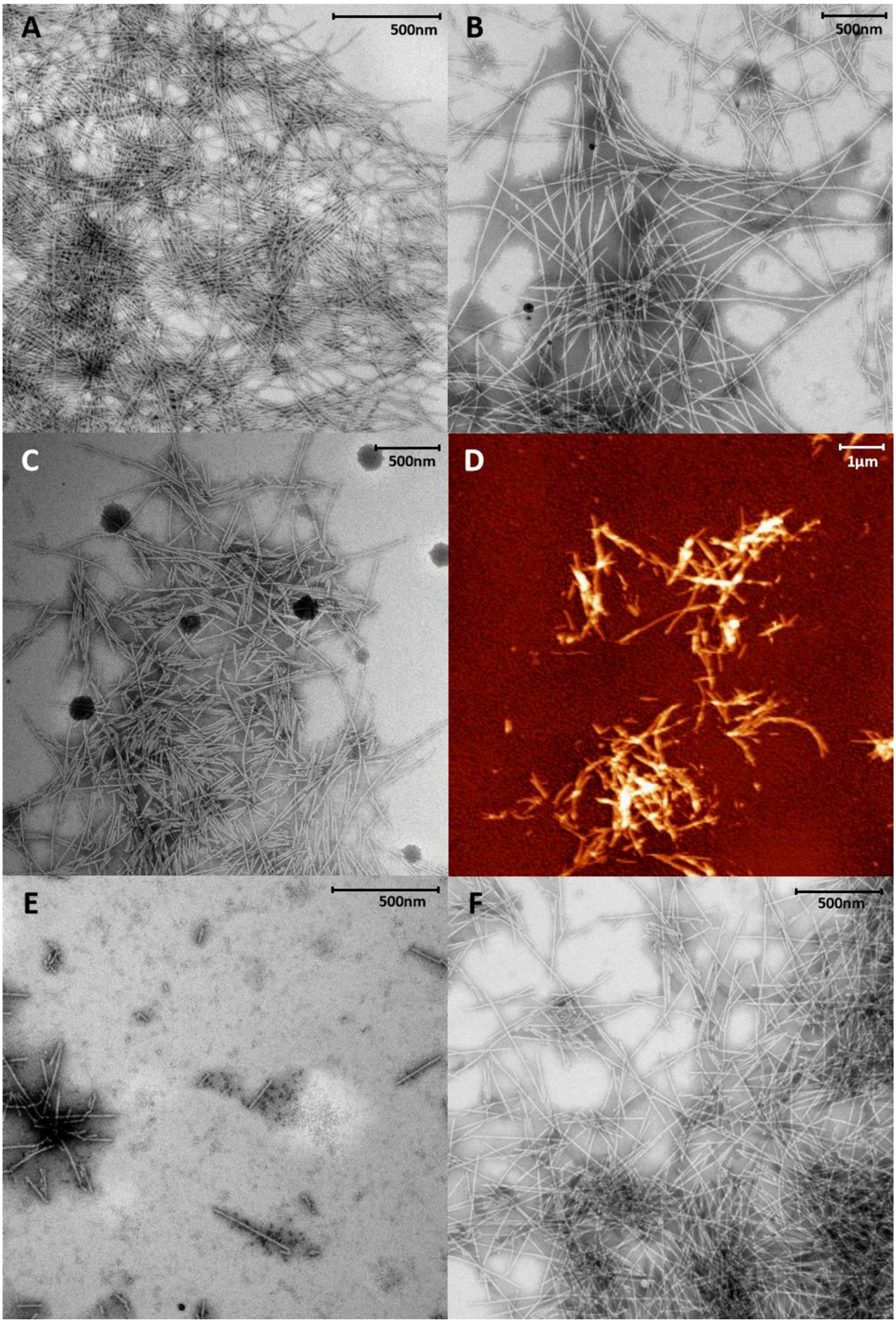
A
500nm
B
500nm
C
500nm
D
1µm
E
500nm
F
500nm

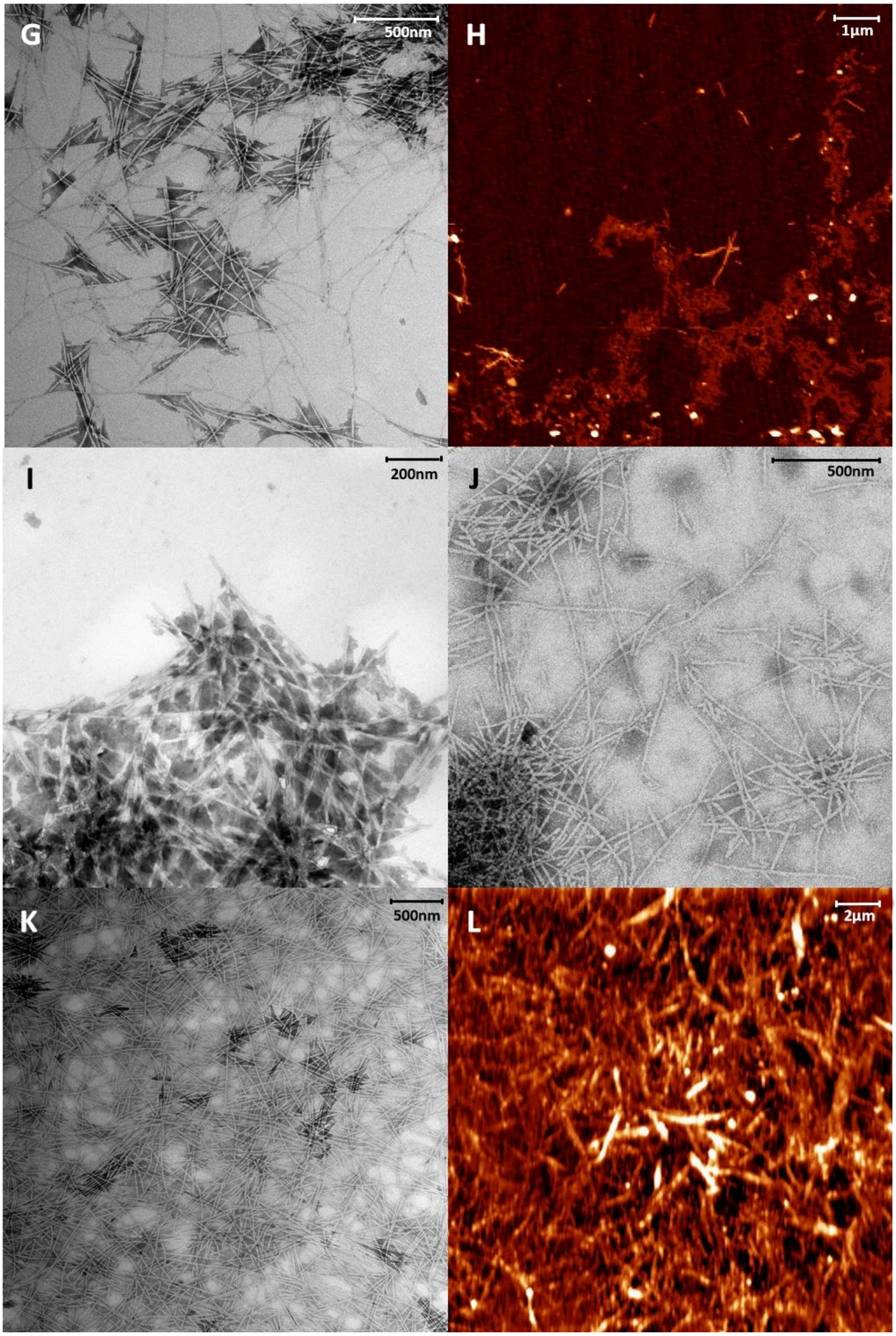

Figure S7. TEM and AFM pictures of the aggregation species formed in the presence and absence of either Cu^{2+} or Fe^{3+}. In order: αSyn A53T (A), with Cu^{2+} (E), and with Fe^{3+} (I). αSyn A53T Ac (B), with Cu^{2+} (F), and Fe^{3+} (J). αSyn WT Ac (C), with Cu^{2+} (G), and Fe^{3+} (K). AFM of αSyn WT (D), with Cu^{2+} (H), and Fe^{3+} (L). Note, because of instrument availability issues, we combined TEM and AFM to have images of each condition.
